# CD133 in Breast Cancer Cells: More than a Stem Cell Marker

**DOI:** 10.1155/2019/7512632

**Published:** 2019-09-16

**Authors:** Federica Brugnoli, Silvia Grassilli, Yasamin Al-Qassab, Silvano Capitani, Valeria Bertagnolo

**Affiliations:** ^1^Department of Morphology, Surgery and Experimental Medicine, University of Ferrara, Ferrara, Italy; ^2^College of Medicine, University of Baghdad, Baghdad, Iraq

## Abstract

Initially correlated with hematopoietic precursors, the surface expression of CD133 was also found in epithelial and nonepithelial cells from adult tissues in which it has been associated with a number of biological events. CD133 is expressed in solid tumors as well, including breast cancer, in which most of the studies have been focused on its use as a surface marker for the detection of cells with stem-like properties (i.e., cancer stem cells (CSCs)). Differently with other solid tumors, very limited and in part controversial are the information about the significance of CD133 in breast cancer, the most common malignancy among women in industrialized countries. In this review, we summarize the latest findings about the implication of CD133 in breast tumors, highlighting its role in tumor cells with a triple negative phenotype in which it directly regulates the expression of proteins involved in metastasis and drug resistance. We provide updates about the prognostic role of CD133, underlining its value as an indicator of increased malignancy of both noninvasive and invasive breast tumor cells. The molecular mechanisms at the basis of the regulation of CD133 levels in breast tumors have also been reviewed, highlighting experimental strategies capable to restrain its level that could be taken into account to reduce malignancy and/or to prevent the progression of breast tumors.

## 1. Introduction

**Figure 1:**
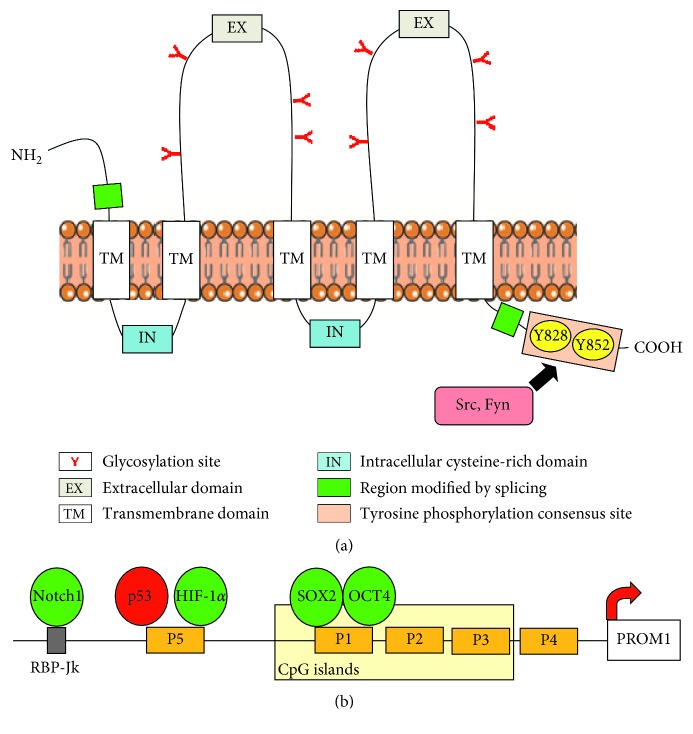
Structure and regulation of CD133. (a) CD133 protein structure in which the C-terminal tyrosine-phosphorylation consensus site, which comprises 5 tyrosine residues including Y828 and Y852, and the splice variants regions are indicated. (b) Schematic representation of the 5′ untranslated region of the CD133 gene. Transcription factors that positively (green circles) or negatively (red circles) regulate CD133 expression by direct binding to the different promoters are reported. The direct binding of Notch1 to the site for RBP-Jk located upstream P1–P5 promoters is also indicated.

CD133/prominin 1 (PROM1) is a pentaspan transmembrane single-chain glycoprotein (Figure 1(a)) mainly localized into protrusions of cellular plasma membrane and particularly in the cholesterol-based lipid microdomains, indicative of its involvement in membrane organization [1]. Transcription of human CD133 is driven by five tissue-specific promoters, three of which located in CpG islands and partially regulated by methylation (Figure 1(b)), leading to spliced mRNAs which results in CD133 isoforms with possibly distinct roles [2].

CD133 was firstly revealed as the target of a monoclonal antibody directed against the AC133 epitope expressed by a subpopulation of CD34^+^ hematopoietic stem cells from the human fetal liver and bone marrow [3]. Despite the initial correlation of CD133 expression with progenitor cells [4, 5], accumulating evidence demonstrated that this surface antigen also characterizes adult tissues, including mammary gland [6–10]. In normal breast tissue, CD133 is not a stem cell marker and plays a role in morphogenesis, regulating ductal branching and the ratio of luminal to basal cells [10]. Even though CD133 has been variously associated with proliferation, cell survival, and autophagy, in precursors and/or mature cells [11], its exact role is not well defined and a specific ligand was not discovered.

The expression of CD133 is deregulated in various solid tumors; however, despite numerous studies, the role of this surface antigen in tumorigenesis and tumor progression is largely unknown [12]. In particular, it is not clear, and in part controversial, the role of CD133 in breast tumors, the most common malignancy and the second cause of cancer-related death among women in industrialized countries. The aim of this review is to summarize the latest findings about the meaning of CD133 in breast cancer, focusing on its relationship with the malignant evolution of the neoplasia.

## 2. CD133 as a Cancer Stem Cell Marker

Most of the studies in solid tumors have been focused on its use as a surface marker for the detection of cells with stem-like properties (i.e., cancer stem cells (CSCs)) [2, 13]. Due to its more restricted expression compared with other CSC markers such as CD44 and aldehyde dehydrogenase (ALDH), CD133 has long been considered the most rigorous indicator of malignant precursors in different solid tumors, including breast cancer [14].

In breast tumors, the role of CD133 as a CSC marker was firstly demonstrated in cell lines derived from BRCA1-associated murine mammary tumors, in which CD133^+^ cells were shown to have a greater colony-forming efficiency, higher proliferative rate, and greater capability to form tumors in NOD/SCID mice [15]. In human invasive breast cancer cell lines, Croker et al. [16] firstly identified subpopulations of cells expressing CD133 together with the putative CSC markers CD44/CD24 and ALDH. When isolated by fluorescence-activated cell sorting and subjected to functional assays, these subpopulations showed increased growth, colony formation ability, migration, invasion, and induced tumorigenesis and metastasis in mice. In particular, ALDH^high^CD44^+^CD133^+^ cells isolated from MDA-MB-231 and MDA-MB-468 cell lines, displaying a triple negative phenotype (ER-, PR-, and HER2-), showed enhanced malignant/metastatic behavior both *in vitro* and *in vivo* [16]. Furthermore, a subpopulation of CD44^+^CD49^high^CD133/2^high^ cells isolated from ER-negative tumors was demonstrated to be enriched for xenograft-initiating cells capable of giving rise to triple negative and ER-negative/HER2-positive tumors [17], endorsing CD133 as a suitable molecule for the identification of CSCs in the most aggressive subtypes of breast cancer. Indeed, when the expression of CD133 was evaluated in breast tumor cell lines with different phenotypes, a strong variability was found. In fact, the number of CD133^+^ cells ranged between 1 and 10% in claudin-low cells, reached 80% in basal-like cell lines, and were between 1 and 2% in both luminal and HER2^+^ cells, questioning the equivalence between CD133 levels and stem-like properties in breast tumor cells [18]. For this reason, although also recently it was used as the sole marker of CSCs [19], CD133 belongs to a well-known panel of molecules that, when properly combined, can actually identify cells with a stem-like phenotype in breast cancer cell lines and primary tumors with different phenotypes [13, 20].

## 3. CD133 as a Prognostic Marker

Although the data concerning the use of the only CD133 to identify CSCs are contradictory, the majority of the studies so far report for CD133 a significant predictive value [21]. Anyway, since CSCs generally express CD133, the prognostic significance of this surface antigen is generally correlated with the stem-like properties of CD133^+^ cells [13].

The role for CD133 as a prognostic marker in breast cancer was firstly demonstrated by Liu et al., who revealed that high PROM1 expression in invasive ductal carcinoma positively correlates with adverse clinic-pathological factors, as tumor size and lymph node metastasis [22]. More recently, it was demonstrated that both CD133 mRNA and protein expression are important biomarkers for prognosis as they positively correlate with higher tumor grade, occurrence of lymph node metastasis, negative PR and ER and positive HER2 status, advanced TNM stage, and poor overall survival (OS) [23–25]. While both cytoplasmic and membrane CD133 were linked to shorter survival, membrane positivity only seems to confer the worst patient outcome. Furthermore, high membrane expression of CD133 was significantly associated with younger age at diagnosis and premenopausal status [26].

Despite the general relationship between CD133 and breast tumor malignancy, some controversies concern the significance of CD133 in tumors with a triple negative phenotype (TNBC), in which *CD133* is strongly hypomethylated with respect to other breast cancer subtypes [27]. A strong negative correlation of CD133 levels with clinical stage of TNBC tumors was firstly observed by Zhao et al. [28], and the use of CD133 to detect circulating tumor cells in TNBC patients ratified its role in prognosis of this breast cancer subtype [29]. Still in TNBC, Cantile et al. suggested that poor prognosis is possibly due to a nuclear mislocalization of CD133, which normally shows a membrane, and more sporadically cytoplasmic, localization [30]. In contrast to all the previous experimental evidences, Collina et al., who described a prevalent cytoplasmic expression of CD133, failed to reveal statistical association of CD133 expression with TNBCs patients' survival [31]. This discordance may be at least in part ascribed to the well-known problem that concerns the different antibodies used to detect CD133 by cytofluorimetrical and immunohistochemical investigations [21], as well as to the absence of standardized criteria to define the scores used for the quantification of the glycoprotein at membrane, cytoplasm, and nuclear level.

A recent study performed with the Gene Expression-Based Outcome for Breast Cancer Online (GOBO) algorithm confirmed that CD133 mRNA is associated with distant metastasis-free survival (DMFS) in patients with all the subtypes of breast cancer [24]. More recently, the overexpression of both CD133 mRNA and protein were investigated in large well-characterized BC cohorts, resulting particularly high in TNBC and HER2^+^ tumors and confirming the negative prognostic value of CD133 in all breast tumor subtypes [26].

In breast cancer, CD133 is also useful in predicting chemosensitivity to neoadjuvant chemotherapy (NAC) [32]. Interestingly, the treatment with NAC resulted in the enrichment of CD133^+^ cells and in the positive correlation of the surface antigen with prognosis, contrarily to its negative significance in pre-NAC tumors [32]. The potential role of CD133 as a marker of chemoresistance in nonluminal breast cancer subtypes was also proposed, on the basis of the relative enrichment of CSCs expressing the surface antigen after systemic therapy [29].

### 3.1. CD133 Regulates Invasive Potential of TNBC-Derived Cells

Various signaling pathways, all directly involved in the acquisition of malignant properties, have been correlated with CD133 levels in solid tumors, supporting its role in different stages of cancer development, including initiation, progression, and metastasis [12]. The identification of CD133 as a substrate for Src and Fyn families of tyrosine kinases suggests that its cytoplasmic domain could play an important role in the regulation of its functions (Figure 1(a)). In particular, the phosphorylation of tyrosine-828 and tyrosine-852 may regulate interaction of CD133 with SH2-domain containing proteins, which may be involved in a number of intracellular signaling events [33], including the activation of PI3K/Akt pathway [34–37].

**Figure 2:**
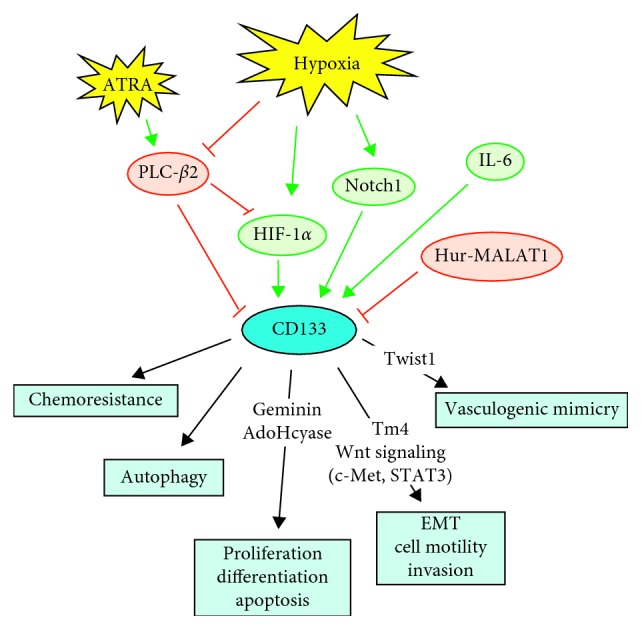
Regulation and functional roles of CD133 in breast cancer. Schematic summary of the main mechanisms regulating CD133 gene expression in breast cancer cells (green circles: positive regulators; red circles: negative regulators) and of cellular events directly targeted by CD133 and involved in breast cancer progression.

At variance with other solid tumors, little is known about the signaling associated to CD133 in breast cancer cells (Figure 2). Interestingly, the almost totality of the data on breast tumors correlate CD133 with molecules involved in cell motility and invasion, suggesting a direct role of PROM1 in modulating the potential malignancy of breast tumors. A role of CD133 in regulating the migration rate of breast cancer cells was firstly revealed in a murine model and involved c-Met and STAT3, both downstream to the Wnt signaling and responsible of cancer invasion and metastasis [38]. A peculiar role of CD133 in the direct modulation of motility and invasive potential of breast tumor cells was demonstrated in the TNBC-derived MDA-MB-231 cell line that comprises a small cellular subset expressing high levels of CD133 at both membrane and cytoplasm levels. Remarkably, the CD133 high cells showed lower proliferation [39], in accordance with the evidence of Di Bonito et al., indicating that only in TNBC, both CD133 mRNA and protein positively correlate with geminin, an inhibitor of cell cycle progression [40]. CD133 high cells also showed a larger adhesion area, consistent with a more differentiated phenotype [39], according to the described role of CD133 in regulating differentiation of normal mammary gland [10]. On the other hand, CD133 high cells exhibited greater invasion capability, suggestive of higher metastatic potential, in accordance with the positive correlation between CD133 and poor prognosis in breast cancer. At variance with other studies, these data highlight the value of both the number of CD133^+^ cells and the expression levels of the surface antigen, which at least in part may justify some discrepancies on the described prognostic role of CD133 in breast tumors.

When MDA-MB-231 subpopulations expressing different levels of CD133 were subjected to two-dimensional electrophoresis followed by mass spectrometry, specific protein signatures were found, including proteins known to be deregulated and to play crucial roles in breast cancer [39]. As expected, the fastest CD133 low cells expressed lower levels of proteins involved in cell cycle and apoptosis, and the most invasive CD133 high cells showed higher expression of proteins with an oncogenic/metastatic role. The CD133-related proteins included the actin-binding protein tropomyosin4 (Tm4), upregulated in highly metastatic breast cancer cell lines and associated with lymph node metastasis of breast tumors [41] and AdoHcyase (Figure 2), known to play a key role in the control of DNA methylation [42] and in the regulation of cell cycle, apoptosis, and cellular differentiation of breast tumor cells [43]. Of note, the silencing of CD133 in CD133 high cells reduced invasiveness and expression of Tm4, ascertaining the existence of direct mechanisms by which CD133 can promote invasiveness of TNBC-derived cells [39].

A relationship between CD133 and EMT markers was demonstrated in tumors cells from metastatic breast cancer patients. In particular, the concomitant overexpression of N-cadherin and CD133 was revealed in both circulating tumor cells [44, 45] and breast cancer specimens [46], even if a significant correlation between the two molecules and patient's prognosis was not fully demonstrated.

## 4. CD133 as a Marker of Malignant Progression Induced by Low Oxygen Availability

A crucial driving force in the progression towards a more aggressive and resistant tumor phenotype is the adaptation of neoplastic cells to a state of reduced oxygen availability defined as hypoxia [47–52]. At least half of all solid tumors, including breast cancer, enclose hypoxic regions varying in amount and size, and recurring tumors often exhibit a hypoxic fraction higher than primary tumors [53]. Intratumoral hypoxia has been identified as an adverse prognostic indicator independent of all the histopathological parameters and, in breast cancer, as in many other solid cancers, low oxygen availability has been reported as associated with a clinically aggressive tumor behavior [54].

In solid tumors, including breast cancer, CD133 is generally induced by low oxygen availability via upregulation HIF-1*α* (Figures 1(b) and 2), even though only in colon cancer cells a physical interaction of HIF-1*α* with the *CD133* promoter was demonstrated [55–58]. Once again, the almost totality of the studies correlating CD133 with low oxygen availability looked at PROM1 as a marker of CSCs, known to increase under hypoxia [50].

In breast tumors, Currie et al. firstly associated the expression of CD133 with markers of hypoxia and/or tumor microvasculature in invasive and noninvasive breast carcinoma [23] although most of the further studies correlating CD133 to low oxygen availability were performed in TNBC. In MDA-MB-231-derived xenografts, CD133^+^ cells with cancer stem cell characteristics were related to vasculogenic mimicry (VM) (Figure 2) and hypoxia induced by the antiangiogenic agent sunitinib [59]. In the same cell model, only CD133^+^ cells formed VM channels in Matrigel after reoxygenation, suggesting that hypoxia accelerates VM by stimulating the CSC population [60]. Again in TNBCs, chemoresistance was associated with higher numbers of CD133/ALDH1 or CD133/CD146 coexpressing cells that were in a quiescent autophagic state related to hypoxia [61]. A further correlation of CD133 with autophagy induced by low oxygen availability was performed in patient-derived TNBC xenografts, in which hypoxia increased drug resistance of CD133^+^ cells, and the inhibition of the autophagic pathway reversed chemoresistance [61].

More recent *in vitro* studies suggest that the effects of hypoxia on the expression of CD133 in breast tumor cells are closely related to their phenotype, and particularly to their ER status. In fact, low oxygen availability seems to induce CD133 only in ER^+^ cells and mostly in cells belonging to the luminal A subtype [62]. At variance with experiments in which hypoxia was pharmacologically induced in xenografts [60], no significant modifications of CD133 were revealed in TNBC-derived cells cultured under low oxygen [62]. At the basis of this discrepancy could be the change of the glycosylation status of CD133 induced by hypoxia, in turn responsible of abnormal detection of the extracellular glycosylated AC133 epitope, as observed in glioma cells [63].

Since hypoxia improves both the number of cells expressing high levels of CD133 and the malignant potential of the noninvasive MCF10DCIS cells [64], the increase of CD133 was considered a marker of malignant evolution induced by low oxygen availability in both noninvasive and low-invasive breast tumors.

## 5. Regulation of CD133 Levels

The expression of CD133 is controlled by many extracellular and intracellular agents, and hypoxic tumor microenvironment and mitochondria dysfunctions seem to be the main events modulating CD133 levels [2, 11]. In particular, hypoxia can improve the levels of the CD133 mRNA by acting at transcriptional level and can increase the recovery of the AC133 epitope, by regulating its glycosylation status [58, 63].

Apart from the hypoxia-related role of HIF-1*α*, there is a general agreement that the transcription factors that interact with CD133 promoters are tumor dependent [12]. For this reason, although substantial evidence assigns to the increase of CD133 levels a crucial role in the malignant potential of various solid tumors, the regulatory mechanisms that promote CD133 expression are still largely unknown in breast cancer. The relationship between CD133^+^ cancer stem cells and the Notch signaling was shown in several tumors, including breast cancer [65, 66], but only in gastric cancer cells, the direct binding of Notch 1 with the promoter region of *CD133* was demonstrated [67]. In colon cancer and osteosarcoma, CD133 expression is negatively regulated by direct binding of p53 to a noncanonical p53-binding sequence in the *CD133* promoter [68]. Moreover, TGF*β*1 is able to regulate CD133 expression in hepatocellular carcinoma through inhibition of DNMT1 and DNMT3*β* expression [69] (Figure 1(b)).

Abnormal DNA methylation, usually reported in many human cancers, seems to play a critical role in CD133 expression, and deregulation of the methylation status was proposed to be at the basis of increased CD133 expression in breast cancer. In particular, D'Anello and colleagues [70] reported that IL-6 induced loss of methylation at *CD133* promoter enhancing CD133 gene transcription in basal-like breast cancer via an autocrine loop triggered by the inactivation of p53. Moreover, in cells with a luminal A phenotype, but not in TNBC-derived cells, the expression of CD133 was linked to MALAT1, one of the most widely studied long coding RNA in cancer development and progression, and to the RNA binding protein HuR (Figure 1(b)). HuR/MALAT1 impact on CD133 gene expression can regulate EMT features, suggesting that the specific regulation of these molecules could control, at least in part, the CD133-related tumor progression [71].

### 5.1. PLC-*β*2 Regulates CD133 in Breast Cancer Cells

An unexpected role in the regulation of CD133 mRNA in breast tumor cells was reported for the beta-2 isoform of PLC (PLC-*β*2) (Figure 2), poorly expressed in normal breast tissues and upregulated in tumor cells, in which sustains motility of invasive cells [72, 73]. The first evidence of a direct regulation of CD133 by PLC-*β*2 was obtained in MDA-MB-231 cells, in which overexpression of the PLC significantly reduced both membrane-associated and cytoplasmic levels of CD133, in parallel with the CD133-related invasion capability [46]. In the same cell model, PLC-*β*2 regulates the amount of CD133^+^ cells with stem-like features. In particular, overexpression of PLC-*β*2 reduced the number of CD44^+^/CD133^+^/EpCAM^+^ cells and proliferation and invasion capability of the CD133^+^/EpCAM^+^ cellular subset [74].

A role of PLC-*β*2 in modulating CD133 expression was also demonstrated in breast tumor derived cells under hypoxia. In particular, culture at low oxygen availability reduced PLC-*β*2 amount and increased CD133 expression in ER^+^ breast tumor cells. Counteracting the decrease of PLC-*β*2 prevented the increase of CD133 induced by hypoxia and significantly reduced the hypoxia-related accumulation of HIF-1*α* (Figure 2), a putative regulator of CD133 in this cell model [62]. The same study demonstrated that PLC-*β*2 is not modified by hypoxia in TNBC-derived cells, in which low oxygen availability fails to induce CD133. On the other hand, its forced expression induced a decrease of the number of CD133^+^ cells, confirming, also in this breast tumor subtype, the role of PLC-*β*2 in downregulating CD133 [62].

PLC-*β*2 is ectopically expressed and regulates the number of cells expressing CD133 also in the noninvasive MCF10DCIS cells [75]. In the same cell model, the administration of all *trans* retinoic acid (ATRA), currently used in the management of acute promyelocytic leukemia [76] in which it induces the expression of PLC-*β*2 [77], counteracts the effects of hypoxia on CD133 expression by up-modulating the PLC isozyme [64]. These data constitute the first evidence that CD133 levels can be modulated by acting on specific signaling molecules and suggest that agonists able to upmodulate PLC-*β*2 could counteract the CD133-related malignant properties in noninvasive and invasive breast tumor cells.

## 6. Conclusion

This review collects the data concerning the expression of CD133 in breast cancer in which this surface antigen is generally associated with a stem cell-like phenotype. In parallel with the role as a cancer stem cell marker, we reviewed the value of CD133 as a prognostic factor and indicator of malignant progression of breast tumors, highlighting its direct role in modulating invasive potential of breast tumor cells with a triple negative phenotype. We also revised the mechanisms regulating CD133 gene expression in both noninvasive and invasive breast tumor cells, underlining experimental strategies capable to limit its expression level that could constitute the basis for new therapeutic approaches to reduce malignancy and/or to prevent progression of breast tumors.
